# Giant bulla or pneumothorax: How to distinguish

**DOI:** 10.1016/j.ijscr.2019.08.003

**Published:** 2019-08-08

**Authors:** Beatrice Aramini, Ciro Ruggiero, Alessandro Stefani, Uliano Morandi

**Affiliations:** Division of Thoracic Surgery, Department of Medical and Surgical Sciences for Children and Adults, University of Modena and Reggio Emilia, Via Largo del Pozzo n. 71, 41124, Modena, Italy

**Keywords:** Pneumothorax, Giant bulla, Chest drain, Bullectomy

## Abstract

**Background:**

The differential diagnosis between pneumothorax and giant bullae is thought to be straightforward but sometimes poses a challenge.

**Case presentation:**

We present a case of a 54-year-old Caucasian man with a giant emphysematous bulla who underwent surgical resection. He had no smoking history and had previous pneumonia episodes. The surgery was free of complications, without air leaks, and he showed good ventilation of the lung.

**Discussion:**

The main complications of bullae are pneumothorax, infection and hemorrhage. Pneumothorax is a serious complication in patients with compromised lung function. Therefore, it is very important to carefully distinguish bullae from pneumothorax to avoid iatrogenic pneumothorax in patients with bullous disease.

**Conclusion:**

We emphasize how to differentiate between giant bullae and pneumothorax utilizing history, physical examination, and radiological studies, including computed tomography (CT) scan.

## Highlights

• The differential diagnosis between pneumothorax and giant bulla is not straightforward.
• We emphasized how to differentiate between giant bulla and pneumothorax.
• The complexity to differentiate induces to make mistakes in diagnosis and in treatment decision.
• Chest CT is mandatory in case of the impossibility to understand the radiological findings.

## 1 Background

The differentiation between pneumothorax and a giant bulla can be very difficult and often leads to inaccurate diagnosis and management. This case report demonstrates the clinical and radiological presentation of giant bullae and highlights the difficulty making a diagnosis and treating appropriately. In particular, we emphasized how to differentiate between giant bullae and pneumothorax through history, physical examination, and radiological assessments, especially computed tomography (CT) scan. This work has been reported in line with the SCARE criteria [1].

## 2 Case presentation

**Fig. 1:**
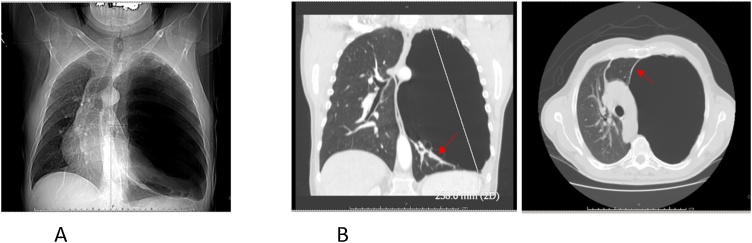
**A.** Preoperative chest X-ray; **1B** Chest-CT showing a giant bulla on the left side, displacing the mediastinum on the right. Arrows indicate the double-wall signs.

A 54-year-old man was referred to the outpatient clinic of our Division for surgical resection of giant emphysematous bullae. He had no smoking history, and he had previous pneumonia episodes and recently experienced intermittent shortness of breath associated with presyncope episodes. Seven days previously, dyspnea worsened (MRC Grade IV) [2]. On general inspection, the patient was alert and stable. A review of his medical record showed a progressive decline in lung function with forced expiratory volume in one second (FEV_1_) that was 65% of the value predicted 6 months previously and FEV_1_ of 1.19 L that was 50% of that predicted 6 months previously. Arterial blood gas showed a pH of 7.41, partial pressure of CO_2_ (PCO_2_) of 43 mmHg, and partial pressure of oxygen (PO_2_) of 69 mmHg. The left hemithorax was hyperresonant to percussion, and decreased breath sounds were apparent upon auscultation. The chest radiography showed progressive enlargement of the bulla in the left lung of over 10 years (Fig. 1A). A CT scan obtained during the current admission revealed a giant bulla with a diameter of 10.5 × 11 cm in the left lobe, causing significant compression of fairly normal lung parenchyma (Fig. 1B).

Surgical criteria for resecting bullae are generally based on symptoms, particularly poor pulmonary function, inducing dyspnea. Moreover, the grade of dyspnea was previously classified [3]: grade I is minimal dyspnea on running or on exerting more than an ordinary effort, grade II is dyspnea on ordinary effort, grade III is considerable dyspnea on exerting less than an ordinary effort, and grade IV is dyspnea at rest. In our case, the patient was of grade II.

**Fig. 2:**
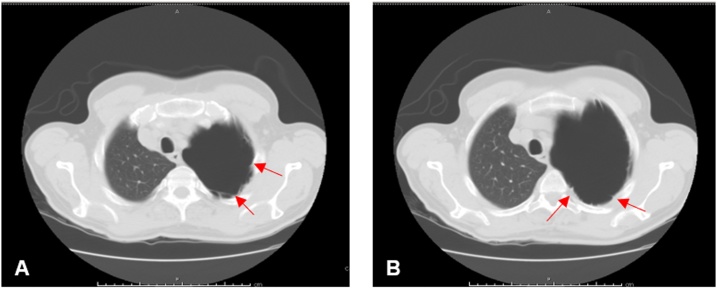
**A–B.** Dense pleural adherences visible on chest CT (arrows).

**Fig. 3:**
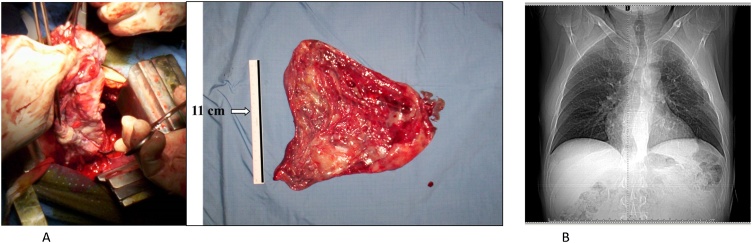
**A.** Surgical isolation of the giant bulla. The bulla measured 11 cm in length. **3B** Six-day postoperative chest X-ray.

The patient underwent surgical resection of the bulla firstly by VATS approach, converted after 30 min into a left thoracotomy for dense pleural adhesions (Fig. 2A and B). The bulla presented as a sessile plant 8–10 cm in length from the apical segment of the upper lobe to the lower lobe of the lung, and it appeared to be of normal consistency. We proceeded to deflate and mobilize the bulla from the parietal pleura and mediastinal adhesions. Bullectomy section was performed by GIA Stapler-75 and reinforced with interrupted sutures of Vycril 2/0 (Fig. 3A). Lysis of the pulmonary ligament was performed. The patient did well postoperatively. Air leakage from the left chest tube stopped on the 3rd postoperative day, and the chest tube was removed. The follow-up chest X-ray showed no pneumothorax, and the patient was discharged in good general clinical condition on the sixth day (Fig. 3B).

## 3 Discussion and conclusion

A “bulla” is defined as an emphysematous space in the lung with a diameter of more than 1 cm in the distended state [4]. Giant bullous emphysema, originally described by Burke [5] in 1937, is an idiopathic, distinct clinical syndrome of severe progressive dyspnea caused by extensive, predominantly asymmetric upper lobe bullous emphysema, which may eventually lead to respiratory failure. Giant bullous emphysema has also been called vanishing lung syndrome [5]. The radiographic criteria for giant bullous emphysema, as defined by Roberts et al. [6], include the presence of giant bullae in one or both upper lobes, occupying at least one third of the hemithorax and compressing the surrounding normal lung parenchyma. Stern et al. [7] described the CT findings of giant bullous emphysema, which include multiple large bullae, ranging from 1 to 20 cm in diameter (usually 2–8 cm), without a single dominant giant bulla. Radiologically, bullae appear as avascular radiolucent areas with thin curvilinear walls. The wall is usually less than 1 mm in thickness and may even be invisible, making detection of the bullae difficult; they are sometimes mistaken for pneumothorax. CT scans are more sensitive than chest x-rays to detect bullae for the accurate assessment of the number, size, and position of bullae, especially when the bullae are obscured [7]. Eligible patients include those with a giant bulla occupying one-fourth or more of one hemithorax on preoperative imaging.

We describe in this report a valuable sign to distinguish pneumothorax from adjacent giant bullae: the double-wall sign. This sign occurs when one sees air outlining both sides of the bulla wall parallel to the chest wall (Fig. 1A and B). The absence of this sign provides further evidence and increased confidence against the diagnosis of pneumothorax, which can prevent unnecessary chest tube placement. The double-wall sign may not be evident on all CT slices, particularly with compression of adjacent bullae, but careful observation of multiple images will reveal this sign when a pneumothorax is present. One potential pitfall in the appreciation of the double-wall sign of pneumothorax occurs when two large bullae are adjacent to one another (Fig. 1B). This situation can produce an apparent double-wall sign, mimicking pneumothorax. However, careful scrutiny of multiple images will show the absence of air in the pleural space and that the bulla wall is not parallel to the chest wall or parietal pleura. The main complications of bullae are pneumothorax, infection and hemorrhage. Pneumothorax is a serious complication in patients with compromised lung function. Therefore, it is very important to carefully distinguish bullae from pneumothorax to avoid iatrogenic pneumothorax in patients with bullous disease.

## Funding

No funding.

## Ethical approval

For single case report NO ethical approval needs. Patient signed a consent for publishing the case report.

## Consent

Patient signed a consent for the publication of this case report.

## Author contribution

BA and CR wrote the case report. AS and UM revised the case report.

## Registration of research studies

Ethical Board approval is not required for case reports in our Center.

## Guarantor

Prof. Uliano Morandi is the Guarantor of this case report.

## Provenance and peer review

Not commissioned, externally peer-reviewed.

## Declaration of Competing Interest

The authors have no financial and personal relationships to disclose.
